# Comment on Shah et al. Genetic Characteristics and Phylogeographic Dynamics of Lagoviruses, 1988–2021. *Viruses* 2023, *15*, 815 †

**DOI:** 10.3390/v16060927

**Published:** 2024-06-07

**Authors:** Joana Abrantes, Stéphane Bertagnoli, Patrizia Cavadini, Pedro J. Esteves, Dolores Gavier-Widén, Robyn N. Hall, Antonio Lavazza, Ghislaine Le Gall-Reculé, Jackie E. Mahar, Stéphane Marchandeau, Ana M. Lopes

**Affiliations:** 1 CIBIO, Centro de Investigação em Biodiversidade e Recursos Genéticos, InBIO Laboratório Associado, Campus de Vairão, Universidade do Porto, 4485-661 Vairão, Portugal; jabrantes@cibio.up.pt (J.A.); pjesteves@cibio.up.pt (P.J.E.); 2 BIOPOLIS Program in Genomics, Biodiversity and Land Planning, CIBIO, Campus de Vairão, Universidade do Porto, 4485-661 Vairão, Portugal; 3 Departamento de Biologia, Faculdade de Ciências, Universidade do Porto, 4099-002 Porto, Portugal; 4 Laboratoire Interactions Hôtes-Agents Pathogènes, Université de Toulouse, INRAE, ENVT, CEDEX 3, 31076 Toulouse, France; stephane.bertagnoli@envt.fr; 5 Istituto Zooprofilattico Sperimentale della Lombardia e dell’Emilia Romagna, 25124 Brescia, Italy; patrizia.cavadini@izsler.it (P.C.); antonio.lavazza@izsler.it (A.L.); 6 WOAH Reference Laboratory for Rabbit Haemorrhagic Disease, Via Bianchi 7/9, 25124 Brescia, Italy; 7 CITS—Center of Investigation in Health Technologies, CESPU, 4585-116 Gandra, Portugal; 8 Swedish Veterinary Agency (SVA), 75189 Uppsala, Sweden; dolores.gavier-widen@sva.se; 9 Department of Biomedical Sciences and Veterinary Public Health, Swedish University of Agricultural Sciences (SLU), Box 7028, 75007 Uppsala, Sweden; 10 Commonwealth Scientific and Industrial Research Organisation, Health and Biosecurity, Canberra, ACT 2601, Australia; robyn.hall@ausvet.com.au; 11 Centre for Invasive Species Solutions, Bruce, ACT 2617, Australia; 12 Ausvet Pty Ltd., Canberra, ACT 2617, Australia; 13 Ploufragan-Plouzané-Niort Laboratory, Avian & Rabbit Virology, Immunology & Parasitology Unit, French Agency for Food, Environmental and Occupational Health and Safety (Anses), 22440 Ploufragan, France; ghislaine.legall-recule@anses.fr; 14 Commonwealth Scientific and Industrial Research Organisation, Australian Animal Health Laboratory and Health and Biosecurity, Geelong, VIC 3220, Australia; Jackie.mahar@csiro.au; 15 French Biodiversity Agency (OFB), 94300 Nantes, France; stephane.marchandeau@ofb.gouv.fr; 16 UMIB—Unit for Multidisciplinary Research in Biomedicine, ICBAS—School of Medicine and Biomedical Sciences, University of Porto, 4050-313 Porto, Portugal; 17 ITR—Laboratory for Integrative and Translational Research in Population Health, 4050-600 Porto, Portugal

**Keywords:** *Lagovirus europaeus*, phylogeny, recombination, evolutionary analysis

Shah and colleagues [1] published an article in *Viruses* where they performed a phylogenetic and phylogeographic analysis of full-length nucleotide sequences of lagoviruses (N = 240). The analysis included pathogenic and non-pathogenic forms of typically rabbit-associated lagoviruses (rabbit hemorrhagic disease virus, RHDV, and rabbit calicivirus, RCV, both belonging to GI according to the nomenclature proposed by Le Pendu et al. [2]) and hare-associated lagoviruses (European brown hare syndrome virus, EBHSV, and hare calicivirus, HaCV, both assigned to GII). According to the authors, the goal of the study was to provide “an update of the phylogenetic and phylogeographic information of lagoviruses that may be used to map the evolutionary history and provide hints for the genetic basis of their emergence and re-emergence”. In our opinion, and despite the authors’ effort in contributing to the current knowledge of lagovirus evolution, some of the methodologies used in the analysis were inappropriate, leading to incorrect and misleading inferences.

Recombination is a major evolutionary force, well documented to occur at a high frequency in some RNA viruses [3,4,5]. It often results from the viral polymerase switching to a new template during recombination, frequently close to the original position due to sequence similarity/homology [6]. This reshuffling of viral genomes results in a mix of genes with different evolutionary histories, and the new genomic combinations might confer novel features to the resulting virus, such as higher pathogenic potential, evasion from the host immune system, resistance to antiviral therapies and vaccines, the expansion of host range (species jump), or the modification of cell or host tropism. As in other RNA viruses, recombination has been described for lagoviruses, with a major recombination hotspot located at the boundary between the non-structural and structural gene-encoding regions and other not-so-frequent breakpoints scattered throughout the genome e.g., [7,8,9,10,11,12,13]. Notably, recombination seems to have contributed to the emergence of GI.2 in 2010 by combining the non-structural proteins of a non-pathogenic GI.3 [2] with the structural proteins of a novel virus, GI.2 [14]. This novel virus was also shown to have recombined with pathogenic GI.1a and GI.1b, as well as non-pathogenic GI.4 [10,15], all contributing to the pool of circulating lagoviruses. Hares have also been a source of new viruses, with both pathogenic and non-pathogenic GII recombining with GI.2 [7,13]. Nonetheless, recombination is not restricted to this genotype, and other key examples of the importance of this mechanism in lagoviruses include the combination of non-pathogenic with classical pathogenic strains, such as the GI.4eP-GI.1a and the GI.3P-GI.1d [12,16] that were able to spread and cause outbreaks in Australia and Europe, respectively.

Evolutionary analyses on recombinant lagoviruses conducted so far considered the impact of recombination in phylogenetic inference. Indeed, in recombinant strains, as the genomic regions on either side of a recombination breakpoint have different evolutionary origins, their phylogenetic history needs to be inferred independently [17,18]. A single phylogenetic tree inferred using the full genome sequence will not reflect the true evolutionary history of a recombinant strain, and no robust conclusions on branch length or topology can be drawn from such analysis. The impact of recombination on tree topology is robustly demonstrated in a recent paper by Zaman and colleagues [19] (see Figure 2 of that paper), which concludes that a reliable phylogeny can only be inferred from a non-recombinant or minimally recombinant region. Moreover, this finding has been thoroughly demonstrated in previous studies [17,20,21], including for other caliciviruses [22], and has motivated, for instance, the currently accepted classification for noroviruses [23,24]. This classification accounts for recombination by using a dual typing system to reflect the origin of both the polymerase and major capsid genes (which flank the recombination hotspot), clearly distinguishing wild-type strains from recombinants, which are “distinct epidemiological entities”. This system was further used as the basis of the nomenclature for lagoviruses proposed by Le Pendu et al. [2] that has been broadly adopted by those studying lagoviruses (e.g., [3,14]).

**Figure 1:**
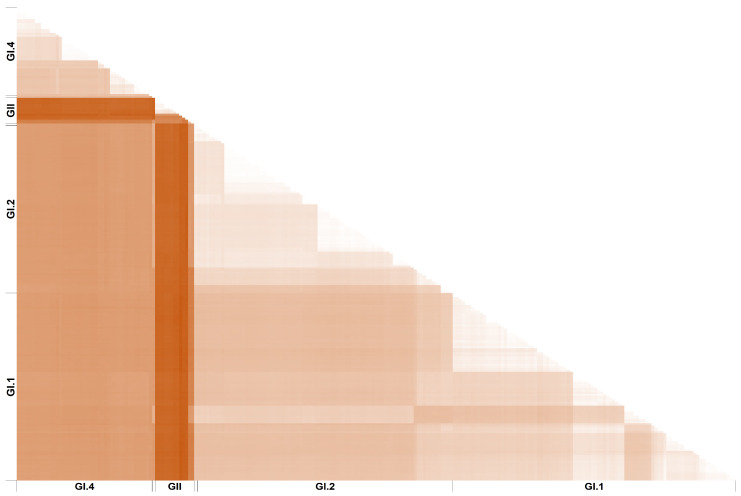
Pairwise genetic distances of the dataset used by Shah et al. [1] (n = 240 sequences, 7337 nucleotides), calculated in MEGAX [48]. Distances were calculated as the number of base substitutions per site between sequences. Ambiguous positions were removed from each sequence pair. Darker color corresponds to the highest distances in the analysis, with a range of values between 0 (white) and 40% (dark orange).

While it has been demonstrated that uncommon recombination events might be “accommodated” in phylogenetic inference [25], the high frequency of recombination events throughout lagoviruses evolution, particularly in GI.2, is likely to distort conclusions inferred from a phylogeny based on full-length genomes. In addition, by performing phylogenetic reconstruction with full-length genomes, recombination events, which evidently shape lagoviruses evolution, might be missed, and the complete epidemiological history obscured. Failure to consider recombination when inferring phylogenies also leads to overestimating the number of point mutations and the substitution rate, and the loss of the molecular clock [17], as has likely occurred in Shah et al. Their study presented a scale bar of 30.0 in the maximum likelihood phylogenies presented in Figure 1 (tree based on the full-length genome sequences) and in Supplementary Figures S1–S3 (trees based on full-length genome sequences, VP60 and VP10 coding sequences, respectively). Although the authors fail to indicate what the scale represents, the default assumption is that the scale bar represents 30.0 nucleotide substitutions/site, an unrealistic level of diversity, particularly given the length of the scale bar. This level of phylogenetic distance is incompatible with the current knowledge on RNA viruses’ evolutionary rates of around 10^−3^ to 10^−4^ substitutions/site/year [5,26].

In their work, Shah and colleagues identified six recombination events: one in VP60, one in the non-structural protein RNA-dependent RNA polymerase (RdRp), and four near the boundary between RdRp and the capsid protein [1], the latter four being consistent with the hotspot of recombination previously described [3,7,10,13]. Despite acknowledging the existence of recombination in the dataset and having included additional VP60- and VP10-based phylogenies, their main conclusions were still inferred from the full-length phylogeny, which led to erroneous conclusions. For example, the authors classified the pathogenic isolate CBMad17-1 (GenBank accession number MF407655) as belonging to non-pathogenic RCV, but this virus has been demonstrated to have resulted from a recombination event between the non-structural region of a GI.4 (non-pathogenic) and the structural part of a GI.2 (pathogenic), i.e., GI.4P-GI.2, using the dual typing nomenclature [27]. Likewise, two GII.1P-GI.2 recombinant German variants (GenBank accession numbers LR899142 and LR899187) [7], critically important as the first examples of intergenogroup recombination in lagoviruses, were identified in Shah et al. as belonging to the HaCV/EBHSV cluster (i.e., GII hare-associated cluster). The authors further highlighted that they observed significant differences between the full-length genome and VP60-based phylogenies, in particular, for GI.1d/Ocun/FR/2009/09-03, P95 and GI.2/Ocun/FR/2013/13-165 (GenBank accession numbers MT628290, KJ943791, and MN737112, respectively), which is congruent with their recombinant origin previously described [12,14,28] and disregarded in this paper. On the other hand, several (previously reported) recombinant viruses remained apparently undetected by their methods, such as those associated with the first reported GI.2 outbreaks in 2010 (GenBank accession numbers KM878681, MN737113, MN737114, MN738377 and MN786321) [14] or the Hartmannsdorf isolate (GenBank accession number EF558586) [11,29]. Thus, it is our opinion that it is not correct to conclude (as they did) that “the full-length genomic sequence-based phylogeny provides a more reliable and robust classification of all the lagoviruses”, and, indeed, we vehemently disagree with this statement.

The topology/clustering demonstrated in both the full-genome and VP60 phylogenies and the phylogeographic network analysis in Shah et al. is also a reason for concern. As stated in their phylogeographic analysis results section, “all HaCV/EBHSV strains clustered together and were sharing their ancestor with the G1.1 (RHDV) strains, (…) [and] all the RCV strains clustered with the GI.2 (RHDV2)” strains. Moreover, their maximum likelihood phylogeny grouped GI.4 (RCV) with GII (HaCV/EBHSV). This contradicts previous phylogenetic and phylogeographic inferences that consistently grouped lagoviruses into two main clusters, one comprising GI variants (RHDV and RCV) and the other composed of GII variants (EBHSV and HaCV) [2,7,30]. Indeed, there appears to be an overlap between phylogenetic clustering and biological host, with GII viruses (EBHSV and HaCV) being restricted to hare species and GI viruses (RHDV and RCV) being mostly found in rabbits. GI.2 is the exception as it has a wider host tropism, fatally infecting other European and American leporid genera (*Lepus*, *Sylvilagus,* and *Brachylagus*) [31,32,33,34,35,36], although it first emerged in rabbits (*Oryctolagus cuniculus*) [37]. Nonetheless, hare species seem to remain as dead-end hosts, and rabbits appear to have a higher susceptibility to lethal disease than other leporids, notwithstanding the limited availability of comparative studies [38]. Phylogenetic analysis in countless previous publications from different labs globally clearly confirm the separation between “rabbit lagoviruses” and “hare lagoviruses”, e.g., [2,7,13,30,33,39]. Furthermore, this partition is confirmed by genetic distances between sequences from these groups (Figure 1).

In another example, their GI.2 clade includes viruses recovered from leporids found dead in 1994–1996 (GenBank accession numbers KY765609–11 and KJ943791) [28], long before the emergence of GI.2 in 2010 [40]. The evolutionary history of these four viruses has been robustly examined previously, demonstrating them to be recombinants with GI structural genes and non-structural genes from a distinct, unclassified genetic group [28]. By ignoring previous analyses and their own VP10 phylogeny, the authors accept that GI.2 was circulating in Portugal in the 1990s whilst causing disease outbreaks only about 15 years later, a much less parsimonious explanation than the existence of a GI.xP-GI.1b recombinant with lower epidemiological fitness, causing local infections and being outcompeted to extinction [28,41]. It is noteworthy that, at the time those viruses were collected, the strains circulating in the Iberian Peninsula were mostly GI.1b [42,43,44]. Hence, it is not surprising that recombination with GI.1b structural regions emerged at this time. Other errors that lead to misinterpretations include the wrong assignment of dates or countries (e.g., RHDV-SD was collected in 1989 rather than in 1993, which corresponds to the submission date in the databases, and RHDV pjG-RHDV-DD06 was collected in the UK rather than in Germany, where the laboratory that submitted the sequence was located). Importantly, the authors claim that “the FRG-USA strain (GenBank ID: NC_001543.1) genome isolated in 2000 is haplotype of the FRG-Germany strain (GenBank ID: M67473.1) isolated in 1991”, while, in fact, they are the same isolate, as clearly stated in Meyers et al. (2000) [45]: “the reference sequence was derived from” Meyers et al. (1991) [46]. As reported by Hicks and Duffy, a single misdated taxon can impact the inferences of evolutionary rates [47].

Finally, the criteria proposed by Shah et al. to assign viruses to groups and define variants in the phylogeny based on the full-length genome sequences is unintelligible, and these are critical points that may cause misinterpretation of lagovirus classification to the reader. For example, in Shah et al. [1], GI.1 sequences do not form a monophyletic group in either the full-genome or the VP60 tree. Additionally, in the full genome tree, the GI.2 sequences are not monophyletic. Therefore, the classification system used by the authors is based on random/non-supported clusters that are not congruent with the epidemiology and known evolution of lagoviruses. Further, the “updated classification” system they provide lacks a substantial number of recently published near-complete genome sequences, as they exclude genome sequences that were published without the 5′ and 3′ end primer binding regions. This results in a less comprehensive and incomplete classification system even if the phylogenetic analysis had been conducted correctly.

In our view, at the time of publication, no evidence exists to support that the approach of using genome-wide alignments for maximum likelihood phylogenetic analysis will produce more reliable “hints for the genetic basis of [lagoviruses] emergence and re-emergence”, given the strong history of recombination in lagoviruses. Indeed, we suggest the opposite: that disregarding recombination when constructing phylogenies leads to missed and/or erroneous inferences by implying a shared evolutionary history of genomic regions that have, in fact, evolved separately. Tracing the evolutionary history of viruses is complex and requires accurate detection and consideration of recombination. In short, while full-genome information is highly useful for complete classification and evolutionary analysis, for genus-wide phylogenetic analysis, it is vital that both the structural and the non-structural encoding regions of lagovirus genomes are inferred independently. Ultimately, the work of Shah et al. does not provide “an update of the phylogenetic and phylogeographic information of lagoviruses”; rather, it proposes a new classification system designed on flawed analyses that will only generate confusion around an already complex classification system for these viruses.
